# Functional assessment of the NMDA receptor variant GluN2A ^R586K^

**DOI:** 10.12688/wellcomeopenres.10985.2

**Published:** 2017-04-26

**Authors:** Katie F.M. Marwick, Peter Parker, Paul Skehel, Giles Hardingham, David J.A. Wyllie

**Affiliations:** 1 Centre for Integrative Physiology, University of Edinburgh, Edinburgh, EH8 9XD, UK

**Keywords:** NMDAR, epilepsy, EEG, intellectual disability, electrophysiology, magnesium, conductance, neurons

## Abstract

*Background:* The N-methyl-D-aspartate receptor (NMDAR) is an ionotropic glutamate receptor that has important roles in synaptogenesis, synaptic transmission, and synaptic plasticity. Recently, a large number of rare genetic variants have been found in NMDAR subunits in people with neurodevelopmental disorders, and also in healthy individuals. One such is the GluN2A ^R586K^ variant ( *GRIN2A* ^G1757A^), found in a person with intellectual disability. Identifying the functional consequences, if any, of such variants allows their potential contribution to pathogenesis to be assessed. Here, we assessed the effect of the GluN2A ^R586K^ variant on NMDAR pore properties.

*Methods*: We expressed recombinant NMDARs with and without the GluN2A ^R586K^ variant in *Xenopus laevis* oocytes and in primary cultured mouse neurons, and made electrophysiological recordings assessing Mg ^2+^ block, single-channel conductance, mean open time and current density.

*Results*: The GluN2A ^R586K ^variant was not found to influence any of the properties assessed.

*Conclusions*: Our findings suggest it is unlikely that the GluN2A ^R586K ^variant contributes to the pathogenesis of neurodevelopmental disorder.

## Introduction

The N-methyl-D-aspartate receptor (NMDAR) is an ionotropic glutamate receptor that has important roles in synaptic transmission and synaptic plasticity, and has been implicated in a range of neurological disorders (reviewed in ( Paoletti *et al.*, 2013)). It is remarkable for its high Ca ^2+^ permeability ( MacDermott *et al.*, 1986), requirement for glycine as a co-agonist ( Johnson & Ascher, 1987) and block by Mg ^2+^, which underlies the NMDAR’s voltage-dependence and hence role as a “molecular coincidence detector” ( Mayer *et al.*, 1984; Nowak *et al.*, 1984). NMDARs are heterotetramers comprised of two GluN1 subunits and two others, of which GluN2A and GluN2B are the commonest in the mammalian forebrain ( Monyer *et al.*, 1994; Watanabe *et al.*, 1992). GluN2A is predominantly expressed post-natally in rodents ( Monyer *et al.*, 1994; Watanabe *et al.*, 1992) and humans ( Law *et al.*, 2003), where it is thought to frequently associate with GluN2B subunits to form triheteromeric GluN2A/2B NMDARs (reviewed in Wyllie *et al.*, 2013).

Recently, advances in sequencing technology have allowed a large number of *de novo* and inherited GluN2A mutations to be identified in individuals with a range of neurodevelopmental disorders, including epilepsy, intellectual disability, autism and schizophrenia ( Burnashev & Szepetowski, 2015). Some of these mutations are gene disrupting, likely resulting in haploinsufficiency, and so far all associated with epilepsy. Other mutations are missense, potentially resulting in a NMDAR with altered function. Identifying the functional consequences, if any, of such mutations may allow insight into key mechanistic pathways underlying the neurodevelopmental disorders with which they are associated. However, it is likely that some mutations are not relevant to the disorders of their carriers, leading either to receptors with no altered function or alterations that are easily compensated for. Distinguishing between mutations that may be pathogenic, and those that are coincidental, aids mechanistic understanding, therapeutics, and genetic counselling.

GluN2A ^R586K^ was first identified as being maternally inherited in a person with intellectual disability and either epilepsy or an abnormal EEG ( Endele *et al.*, 2010). The phenotype of the proband’s mother was not reported. GluN2A ^R586K^ has undergone no previous functional assessment. However, *in silico* prediction tools found it to be “possibly damaging” and “non-neutral” ( Endele *et al.,* 2010), albeit it is a conservative, within-class amino acid substitution, with both arginine and lysine being positively charged. The substitution alters a residue in the cytosolic loop linking the subunit’s M1 and M2 pore domains ( Monyer *et al.*, 1992), which has so far not been linked to any particular function. However, the loop’s proximity to the pore suggests that any functional consequences could include an impact on ion permeation and/or block of the pore by Mg ^2+^ ions. In the present study, we performed the first known functional analysis of the GluN2A ^R586K^ variant. We made electrophysiological recordings from NMDARs containing mutant subunits expressed in *Xenopus laevis* oocytes and primary cultured neurons, allowing us to assess the variant’s impact on block by Mg ^2+^ ions, single channel conductance, mean open time and NMDAR current density. We found that the GluN2A ^R586K^ variant did not affect any of the parameters assessed.

## Materials and Methods

### Constructs

The cDNA for wild type human NMDA subunit GluN1-1a (hereafter GluN1) (GenBank accession code NP_015566), GluN2A (GenBank accession code NP_000824; ( Hedegaard *et al.*, 2012)) and GluN2A ^R586K^ were gifts from Dr Kasper Hansen and Dr Honjie Yuan (University of Emory, Atlanta, GA, USA). The GluN2A ^R586K^ variant was confirmed by Sanger sequencing. All cDNAs were expressed via a pCI-neo vector.

### Two-electrode voltage-clamp recordings

cRNA for wild type and mutant subunits was synthesized from linearized plasmid DNA as runoff transcripts using the T7 polymerase mMessage machine RNA synthesis kit (Life Technologies Ltd, Paisley, UK). Each oocyte was injected with 3.7–9 ng of cRNA, comprising a 1:1 molar ratio of GluN1 and GluN2A diluted in RNAse free water.

Stage V-VI *X. laevis* oocytes were obtained from the UK Xenopus centre (Portsmouth, UK) and from Diaclean (CastropRauxel, Germany), collagenased (200 units/ml for 60 minutes), then manually defolliculated prior to injection. After injection, oocytes were placed in separate wells of 24-well plates containing a modified Barth’s solution with composition (in mM): 88 NaCl, 1 KCl, 2.4 NaHCO _3_, 0.82 MgCl _2_, 0.44 CaCl _2_, 0.33 Ca(NO _3_) _2_, 15 Tris-HCl; adjusted to pH 7.35 with NaOH. This solution was supplemented with 50 IU/ml penicillin, 50 mg/ml streptomycin and 50mg/ml tetracycline. Oocytes were placed in an incubator (16–21°C) for 24–48 hours to encourage receptor expression and subsequently stored at 4°C. Recordings were made 48–96 hours post injection.

Two-electrode voltage-clamp recordings were made at room temperature (18–21°C) from oocytes that were placed in a solution that contained (in mM): 115 NaCl, 2.5 KCl, 10 HEPES, 1.8 BaCl _2_, 0.01 EDTA; pH 7.35 with NaOH. Recordings were made using a GeneClamp 500B amplifier (Molecular Devices, Union City, CA, USA). Current and voltage electrodes were made from thin-walled borosilicate glass (GC150TF-7.5, Harvard Apparatus, Kent, UK) using a PP-830 electrode puller (Narashige Instruments, Tokyo, Japan). Filling with 3 M KCl gave resistances of between 0.2 and 1.5 MΩ. Application of solutions was determined manually. Data were filtered at 10 Hz and digitized at 100 Hz via a 1401 plus analogue-digital interface (Cambridge Electronic Design, Cambridge, UK) using WinEDR software (version 3.2.7; Strathclyde Electrophysiology Software, Strathclyde University, Glasgow, UK). Oocytes were voltage-clamped at –60 mV. Recordings were rejected if the holding current (in nA) was greater than three times the holding potential (in mV), or if the holding current drifted by more than 10% of the agonist response across the course of the experiment.

### Single-channel voltage-clamp recordings

Single-channel voltage-clamp recordings were made at room temperature from outside-out patches pulled from oocytes that were placed in a solution that contained (in mM): 125 NaCl, 3 KCl, 1.25 NaH _2_PO _4_, 20 HEPES, 0.85 CaCl _2_, 0.01 EDTA; pH 7.35 with NaOH. Steady state recordings were made in the presence of 30 μM glutamate and 30 μM glycine. Recording durations varied from 30 seconds to 5 minutes. Prior to recording, vitelline membranes were manually removed from oocytes following placement in a hypertonic solution that contained (in mM): 200 sodium methyl sulphate, 20 KCl, 10 HEPES, 1 MgCl _2_; pH 7.4 with NaOH. Recordings were made using an AxoPatch 1D amplifier (Molecular Devices). Electrodes were made using thick walled borosilicate glass (GC150F-7.5; Harvard Apparatus) using a P-87 electrode puller (Sutter Instrument, Novato, CA, USA) and their tips fire polished to give resistances of 7 to 12 MΩ when filled with internal solution containing (in mM): 2.5 NaCl, 141 K-gluconate, 10 HEPES, 11 EGTA; pH 7.4 with KOH. Electrode tips were coated in silicone elastomer (“sylgard 184”; Dow Corning, Wiesbaden, Germany) to reduce capacitance. Application of solutions was controlled manually. Data were prefiltered at 2 kHz (–3 dB; 8th order Bessel filter) and digitized at 20 kHz via a Micro 1401 analogue-digital interface (Cambridge Electronic Design) using WinEDR software (v 3.2.7). Patches were voltage-clamped at –60 or –100 mV.

WinEDR v3.3.7 was used to idealise the traces (using a transition threshold of 50% of the unitary conductance level and a 100 μs open and shut resolution), to fit Gaussian curves to amplitude histograms and to fit exponential curves to dwell time durations (all curves fitted using iterative maximum likelihoods). Any openings where more than one channel was open simultaneously were discarded. Conductance was calculated by dividing the current amplitude by the holding potential.

### Preparation and transfection of neurons

Animal breeding, maintenance and procedures were performed in accordance with the UK Animal Scientific Procedures Act (1986). Culturing was a modified version of ( Furshpan & Potter, 1989). Brains from E17.5 CD1 mice (sex not determined, on average 15 mice per culture) were microdissected in medium containing (in mM): 8.8 Na _2_SO _4_, 27 K _2_SO _4_, 5.3 MgCl _2_, 0.23 CaCl _2_, 0.9 HEPES, 0.001% Phenol Red, 18 D-glucose, 0.0005 kynurenic acid; adjusted to pH 7.35 with NaOH. Cortices were incubated at 37°C for 40 minutes in papain enzyme (36,000 USP units/ml; Worthington Biochemical Corporation, Lakewood, NJ, USA) then washed and triturated in NeuroBasal A medium (supplemented with 1% rat serum (Harlan Laboratories, Bicester, UK), 1 × B-27 supplement, 1% antibacterial/antimycotic and 1 mM glutamine). The cell suspension was diluted in opti-MEM (supplemented with 20 mM glucose and 1% antibacterial/antimycotic) to give an end concentration of 1 hemisphere per 7 ml, and 0.5 ml/coverslip plated onto 13 mm diameter coverslips precoated with poly-D-lysine (1.33% w/v in H _2_0) and laminin (0.5% w/v) (Roche, Basel, Switzerland) in 24-well plates. Plates were kept at 37°C in a humidified 5% CO _2_ incubator for 2.5 hours before replacement of the cell suspension with supplemented NeuroBasal A. On day *in vitro* (DIV) 4, 1 ml/well of supplemented NeuroBasal A containing 9.6 mM cytosine β-D-arabinofuranoside hydrochloride was added to the cells.

Neurons were transfected on DIV 7 or 8 with plasmids containing cDNA for wild type and mutant GluN2A subunits, or the inert control β globin, using Lipofectamine 2000 (hereafter referred to as lipofectamine) in serum free non-trophic transfection medium composed of: 10% minimum essential media (MEM; +Earles, -L-glutamine), 88% Salt-Glucose-Glycine (comprising in mM 114 NaCl, 26 NaHCO _3_, 5.3 KCl, 1 MgCl _2_, 2 CaCl _2_, 10 HEPES, 1 glycine, 30 D-glucose, 0.5 sodium pyruvate, with phenol red 0.001%; ( Bading *et al.*, 1993)) supplemented with 1% antibiotic/antimycotic and 1% insulin–transferrin–selenium supplement. For each cover slip, 2.3 μl of lipofectamine was mixed with 575 ng plasmid DNA comprising a 2:1 mass ratio of GluN2A/GluN2B and eGFP cDNA. Cotransfection rate was 100% [cells were co-transfected using the above technique with cDNA encoding two fluorescent proteins (eGFP and mCherry) and observed for overlapping fluorescence 48 hours post transfection: 47/47 cells observed over 3 cover slips showed co-transfection]. Electrophysiological recordings were made 48 hours post transfection.

### Whole-cell recordings in cultured neurons

Recordings from cultured neurons were made at room temperature with neurons superfused (at a flow rate of 2 mL/min) with external recording solution composed of (in mM) 150 NaCl, 2.8 KCl, 10 HEPES, 2 CaCl _2_, 10 glucose, 0.1 glycine, 0.003 tetrodotoxin; pH 7.35 using NaOH (300–330 mOsm). Transfected cells were identified by eGFP expression (excitation at 470 nm, coolLED pE-100 (coolLED Ltd, Andover, UK)). In total, 150 μM NMDA was applied briefly twice to elicit desensitization, then reapplied until a steady state response was achieved (around 10 seconds of application) at which point 1 mM MgCl _2_ was co-applied until a new steady state was achieved. Experiments were then repeated after perfusion with 3 μM ifenprodil for one minute. Application of solutions was controlled manually. Patch-pipettes were made from thick-walled borosilicate glass (GC150F-7.5; Harvard Apparatus) using a P-87 puller (Sutter Instruments) to give a resistance of 2–4 MΩ when filled with internal solution containing (in mM): 141 K-gluconate, 2.5 NaCl, 10 HEPES, 11 EGTA; pH 7.3 with KOH (300 mOsm). Currents were recorded using an Axopatch 200B amplifier (Molecular Devices). Data were filtered at 2 kHz and digitized at 20 kHz via a National Instruments BNC-2090A analogue-digital interface (National Instruments) using WinEDR software (v 3.2.7). Neurons were voltage-clamped at –65 mV, and recordings were rejected if the holding current was greater than 150 pA or if the series resistance was greater than 30 MΩ, or increased by greater than 20% during the course of the recording. Capacitance was calculated by calculating the area under the current response to a 5 mV test pulse plotted against time (giving the charge) and dividing by the voltage of the test pulse. Capacitance provides an estimate of cell surface area. Current density was then calculated as current/capacitance.

### Data analysis

Bar graphs depict individual cells (circles), means (columns) and standard error of the mean (SEM; error bars). R (v 3.1.2; R Core Team, 2014) was used to perform statistical tests. Comparisons between multiple means were performed by ANOVA. Comparisons between two means were performed by independent, two-tailed, Welch t–tests (which do not assume equal variance between groups), unless otherwise stated. The significance level used was p <0.05, corrected for multiple comparisons using the Bonferroni method. In figures, * indicates corrected significance levels of p <0.05, ** indicates p <0.01 and *** indicates p <0.001.

## Materials

NMDA, NMDAR antagonists, ifenprodil and tetrodotoxin were purchased from Tocris Bioscience (Bristol, UK). Media, media supplements, Lipofectamine 2000, antibiotic/antimycotic and B-27 were purchased from Invitrogen (Carlsbad, CA, USA). The remaining substances were purchased from Sigma-Aldrich (St. Louis, MO, USA) unless stated otherwise in the text.

## Results

### The GluN2A ^R586K^ mutation has no effect on Mg ^2+^ block or current density

**Figure 1.**
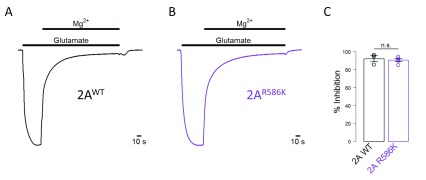
NMDARs containing GluN2A ^R586K^ show normal block by Mg ^2+^ when expressed in oocytes. ( **A** and **B**) Representative two-electrode voltage-clamp recordings from oocytes expressing GluN2A ^WT^ or GluN2A ^R586K^-containing NMDARs, showing current evoked by glutamate (30 μM) and inhibition by an approximately physiological concentration of Mg ^2+^ (1 mM), in the continuous presence of glycine (30 μM). Holding potential –60 mV. Responses are normalised to the peak amplitude. ( **C**) Summary data showing percentage block by Mg ^2+^ at –60 mV. A t-test showed no significant difference between Mg ^2+^ block in oocytes expressing GluN2A ^WT^ (n = 3, 92 ± 3 %) and GluN2A ^R586K^ (n = 5, 90 ± 2 %, t(2.9) = 0.46, p = 0.68).

We first expressed GluN2A ^WT^ and GluN2A ^R586K^–containing NMDARs in *X. laevis* oocytes and made two-electrode voltage clamp recordings of block by Mg ^2+^ of glutamate-evoked currents. We found no effect of GluN2A ^R586K^ on block by Mg ^2+^ ( Figure 1A–C).

To assess for any neuron-specific consequences of the variant, we then used transient transfection to over-express GluN2A ^R586K^ in cultured primary mouse cortical neurons. The interpretation of results in neurons is complicated by each cell giving a NMDA-evoked response that arises in part from endogenous NMDAR subunits and in part from transfected subunits. The age of culture used (DIV 9) was chosen so that virtually all the endogenous GluN2 subunits were GluN2B ( McKay *et al.*, 2012). The GluN2B selective negative allosteric modulator ifenprodil could therefore be used to suppress the contribution of endogenous NMDARs and to confirm the transfection of the subunit of interest (GluN2B diheteromers show 80% block, but GluN2A diheteromers minimal block at the concentration of ifenprodil used (3 μM) ( Williams, 1993)).

**Figure 2.**
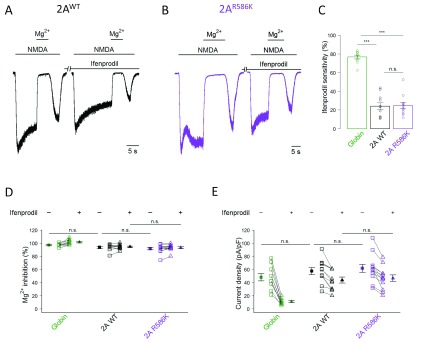
NMDARs containing GluN2A ^R586K^ show normal block by Mg ^2+^ and normal current density when expressed in neurons. ( **A** and **B**) Representative whole-cell voltage-clamp recordings from day *in vitro* (DIV) 9 neurons transfected with an inert control, GluN2A ^WT^ or GluN2A ^R586K^, showing inhibition of current evoked by saturating NMDA (150 μM) by Mg ^2+^ (1 mM) before and after one minute of ifenprodil (3 μM) application. Holding potential –65 mV. Responses are normalised to the peak amplitude. ( **C**) Summary data showing percentage inhibition by ifenprodil, recorded from neurons as shown in **A** and **B**. A one-way ANOVA showed a significant effect of transfected subunit (F(2, 30) = 91, p = 1.8e-13), with *post hoc* t-tests (p _corr_ = 0.15) showing that neurons transfected with GluN2A ^WT^ showed lower ifenprodil sensitivity than control transfection cells (t(15.7) = 12.6, p = 1.3e-9), as did neurons transfected with GluN2A ^R586K^ (t(18.3) = 13.8, p = 3.9e-11), and with no difference between GluN2A ^WT^ and GluN2A ^R586K^ (t(20.3) = 0.2, p = 0.88). These results confirm that the GluN2A ^R586K^ subunits were successfully trafficked to the membrane in neurons. ( **D**) Summary data showing percentage inhibition by Mg ^2+^ in the presence and absence of ifenprodil, recorded from neurons as shown in **A** and **B**. A two-way ANOVA showed a significant effect of transfected subunit (F(2, 30) = 7.5, p = 0.0022) on Mg ^2+^ block and of the presence/absence of ifenprodil (F(1, 30) = 17.1, p = 0.0003), with a significant interaction (F(2, 30) = 4.4, p = 0.021). However, *post hoc* t-tests (p _corr_ = 0.15) showed no difference in Mg ^2+^ block in the absence of ifenprodil in neurons transfected with GluN2A ^R586K^ compared to GluN2A ^WT^ (t(20.3) = 0.86, p = 0.40), and no difference between GluN2A ^WT^ and neurons transfected with an inert control (t(13.8) = 2.2, p = 0.049). There was also no reduction in Mg ^2+^ block associated with GluN2A ^R586K^ vs GluN2A ^WT^ in the presence of ifenprodil, when a greater proportion of response was attributable to the transfected subunits of interest (t(20.2) = 0.87, p = 0.40). ( **E**) Summary data showing current density evoked by NMDA in the presence and absence of ifenprodil, recorded from neurons as shown in **A** and **B**. A two-way ANOVA showed a significant effect of transfected subunit (F(2, 30) = 7.5, p = 0.0023) and of the presence/absence of ifenprodil (F(1, 30) = 139, p = 9e-13), with a significant interaction (F(2, 30) = 16.8, p = 1.3e-5). However, *post hoc* t-tests (p _corr_ = 0.15) showed no difference in current density in the absence of ifenprodil in neurons transfected with GluN2A ^R586K^ compared to GluN2A ^WT^ (t(20.9) = 0.5, p = 0.61), and no difference between GluN2A ^WT^ and neurons transfected with an inert control (t(18.8) = 1.2, p = 0.24). There was also no reduction in current density associated with GluN2A ^R586K^ vs GluN2A ^WT^ in the presence of ifenprodil (t(21) = 0.44, p = 0.66). Data are means ± SEM. Circles represent individual cells. See Table 1 for means and sample sizes.

GluN2A ^R586K^ expression was confirmed by reduced ifenprodil sensitivity compared to control transfections ( Figure 2A–C; Table 1). As with oocytes, no effect was found on Mg ^2+^ block in neurons ( Figure 2A,B,D; Table 1). Current density was also unaffected by the GluN2A ^R586K^ mutation ( Figure 2A,B,E; Table 1).

**Table 1.** Mg ^2+^ block, current density and ifenprodil sensitivity in neurons transfected with an inert control (Globin), GluN2A ^WT^ and GluN2A ^R586K^ subunits.

	Ifenprodil sensitivity	Mg ^2+^ block (%)	Mg ^2+^ block (%) + Ifenprodil	Current density (pA/pF)	Current density (pA/pF) + Ifenprodil	n
Globin	77 ± 2	97 ± 1	101 ± 1	48 ± 6	11 ± 1	10
GluN2A ^WT^	24 ± 4	94 ± 1	94 ± 1	57 ± 5	44 ± 5	11
GluN2A ^R586K^	25 ± 3	92 ± 2	93 ± 1	62 ± 6	47 ± 5	12

n refers to number of neurons. Data are means ± SEM. Statistical comparisons are reported in the Figure 2 legend. Mg ^2+^ concentration used was 1 mM; ifenprodil 3 μM.

### The GluN2A ^R586K^ mutation has no effect on single-channel properties

**Figure 3.**
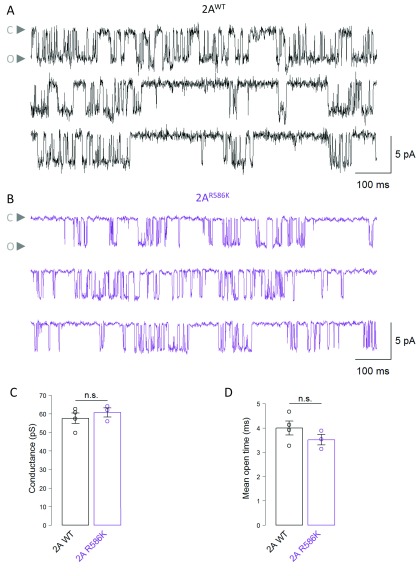
NMDARs containing GluN2A ^R586K^ show normal single-channel conductance. ( **A** and **B**) Representative voltage-clamp recordings made from outside-out patches from oocytes transfected with GluN2A ^WT^ or GluN2A ^R586K^-containing NMDARs in the presence of glutamate (30 μM) and glycine (30 μM). “C” = closed, “O” = open. ( **C**) Summary data showing conductance, calculated as current amplitude/holding potential. A t-test showed no significant difference in conductance in oocytes expressing GluN2A ^WT^ (n = 4, 58 ± 3 pS, total events = 2555) and GluN2A ^R586K^ (n = 3, 61 ± 2 pS, total events = 1139, t(5.0) = 0.84, p = 0.44). ( **D**) Summary data showing tau for mean open time, calculated by fitting exponential curves to open time frequency distributions. A single exponential was fitted in each case. A t-test showed no significant difference in mean open time in oocytes expressing GluN2A ^WT^ (n = 4, 4.0 ± 0.3 ms) and GluN2A ^R586K^ (n = 3, 3.5 ± 0.2 ms, t(5.0) = 1.32, p = 0.24). Data are means ± SEM. Circles represent individual cells.

Finally, we used outside-out patches pulled from oocytes to assess the impact of GluN2A ^R586K^ on NMDAR single-channel conductance and mean open time. We found GluN2A ^R586K^ to have no effect ( Figure 3A–D).

## Discussion

In the present study, we investigated the functional consequences of a GluN2A point variation found previously in a person with epilepsy/EEG abnormalities and intellectual disability. Using heterologous systems, we showed that the GluN2A ^R586K^ variant had no effect on any of the properties assessed: Mg ^2+^ block, current density, conductance or mean open time.

The lack of functional consequences on permeation and Mg ^2+^ block seen with the GluN2A ^R586K^ variant is in contrast to existing findings for several disease-associated NMDAR mutations, which have been found to have a substantial impact on NMDAR properties, including Mg ^2+^ block ( Burnashev & Szepetowski, 2015). This is not unsurprising given the diversity of mutations found in NMDAR subunits, with some resulting in complete loss of function of the allele, some impacting residues of established functional importance, and some causing conservative changes in residues of unknown function, such as studied here.

It was difficult to hypothesise what the likely impact of the GluN2A ^R586K^ variant might be, as little is known about the function of the M1-M2 linker region in which it is found. It is possible that the variant affects other properties that have not been investigated in this study. For example, conceivably it could influence trafficking or zinc affinity, which is known to be regulated by C-terminal phosphorylation ( Zheng *et al.*, 1998). Further, the impact of the sequence change could manifest through indirect mechanisms, such as an impact on mRNA transcript stability, or epigenetic modifications. Finally, a given variant may only have a physiological effect when partnered with a further variant (compound heterozygosity). In general, it is impossible to exclude that a variant has some functional effect.

However, it is certainly possible that the variant does not alter NMDAR function, and that its presence in an individual with neurodevelopmental disorder was coincidental. This is particularly likely as the variant was inherited, and because it has also subsequently been found in two out of 60,706 individuals without severe paediatric disease collated by the Exome Aggregation Consortium ( Lek *et al.*, 2016) - although the existence of less severe neurodevelopmental disorders, such as adult onset epilepsy, in these individuals has not been excluded.

In conclusion, our limited functional studies suggest that the GluN2A ^R586K^ variant is probably benign.

## Data availability

Data are deposited in project GluN2A R586K, Open Science Framework: DOI, 10.17605/OSF.IO/AWCNQ ( Marwick, 2017).
