# Supplementary material for: Functional assessment of the NMDA receptor variant GluN2A R586K
Source: Wellcome Open Res. 2017 Apr 26;2:20. Originally published 2017 Mar 17. [Version 2] doi: 10.12688/wellcomeopenres.10985.2 (PMC5407442; doi:10.12688/wellcomeopenres.10985.2)
Supplement: Peer review history

# Peer review: Referee response for version 2

**DOI:** 10.21956/wellcomeopenres.12411.r22270

**Published:** 2017-05-12

**Authors:** Ian R. Mellor, Helen Knight

**Affiliations:** 1 School of Life Sciences, University of Nottingham, Nottingham, UK; 2 University of Nottingham, Nottingham, UK

The authors have address most of the points raised in our original report. We have no further comments.

We have read this submission. We believe that we have an appropriate level of expertise to confirm that it is of an acceptable scientific standard.

# Peer review: Referee response for version 2

**DOI:** 10.21956/wellcomeopenres.12411.r22272

**Published:** 2017-05-02

**Authors:** Laetitia Mony

**Affiliations:** 1 INSERM (French National Institute of Health and Medical Research), Paris, France

The authors have addressed most points I had raised in my first review. While I still think that testing the effect of magnesium at a more depolarized potential, for which magnesium sensitivity is decreased, might have been informative, the authors have brought significant improvement to their introduction and discussion.

 I am therefore in favor of indexing of this article in its current state.

I have read this submission. I believe that I have an appropriate level of expertise to confirm that it is of an acceptable scientific standard.

# Peer review: Referee response for version 1

**DOI:** 10.21956/wellcomeopenres.11843.r21703

**Published:** 2017-04-19

**Authors:** Laetitia Mony

**Affiliations:** 1 INSERM (French National Institute of Health and Medical Research), Paris, France

The current manuscript describes the *in vitro* functional characterization of a GluN2A mutation, GluN2A ^R586K^, found in a patient with intellectual disability and either epilepsy or abnormal EEG ^1^. A subsequent number of mutations within the GRIN1, GRIN2A and GRIN2B genes were found in patients with neurological disorders ^1^^-^^3^. *In vitro* functional characterization revealed that some of these mutations had major impacts on NMDA receptor expression, biophysical and pharmacological properties (see, for example, Swanger et al. and Serraz et al. ^4^^,^^5^). However, although predicted to be “possibly damaging” by Endele et al. ^1^, the functional effect of the GluN2A ^R586K^ mutation had never been investigated. Since R586 is located in the M1-M2 loop, close to the pore, they logically investigated the permeation and block properties of the GluN1/ GluN2A ^R586K^ mutant and found that magnesium block, conductance and mean open times were unaffected in Xenopus oocytes and cultured neurons. They thus concluded that this mutation is probably benign.

 The manuscript is written well and the experiments are clearly described in the experimental section. I have however several reservations concerning the experimental work and some suggestions for the discussion, which I hope might improve the quality of the manuscript.  

1. Magnesium block: The authors only test the effect of one concentration (1mM), at one holding potential. Although 1mM magnesium is close to the physiological concentration, it seems that this concentration is saturating for GluN1/GluN2A receptors at -60 mV, which may hide differences in magnesium sensitivities between wt and R586K receptors. The authors should test different concentrations and different holding potentials to be able to conclude that magnesium block is unaffected by the mutation. Ideally, full current-voltage relationships should be measured at different magnesium concentrations.
2. Pore opening properties: In their single channel experiments, the authors measure the single channel conductance and the mean open time. What about the open probability? Is it also unaffected by the mutation?
3. Limiting the functional studies to the sole permeation and block properties of NMDARs is very restrictive. Although the mutation is located far from the extracellular domains, where most pharmacological agents act, it could very well affect the pharmacological properties of the receptor. For example, it has been shown that phosphorylation of residues located in the intracellular C-terminal tail could influence affinity for zinc, which binds in the extracellular N-terminal domain ^6^. Therefore, to conclude that the mutation has no clear functional effect, the authors should have measured the mutant sensitivity for the agonists glutamate (or NMDA) and glycine, its kinetics of activation and deactivation, as well as its sensitivity for the endogenous allosteric modulators zinc and protons.
4. Relative to point #3, in the discussion section, where the authors state “The lack of functional consequences of the GluN2A ^R586K^ variant”, they should say “The lack of consequences on the permeation and block properties of the GluN2A ^R586K^ variant”. Indeed, the variant could have functional consequences that were not investigated in the study.
5. In the discussion, the authors mention that “It is also possible that the variant affects other properties that have not been investigated in this study”. The authors should discuss in more details what properties could be affected by the variant (as previously mentioned, pharmacological properties, but also trafficking, downstream signaling…).
6. Relative to points #3-5, the authors can certainly conclude that the mutation does not induce any dramatic effect on NMDAR function, which would show even at saturating agonist and magnesium concentrations. However, to conclude that it is “probably benign” would require further characterization of this variant.

I have read this submission. I believe that I have an appropriate level of expertise to confirm that it is of an acceptable scientific standard, however I have significant reservations, as outlined above.

## References

[1] EndeleSRosenbergerGGeiderKPoppBTamerCStefanovaIMilhMKortümFFritschAPientkaFKHellenbroichYKalscheuerVMKohlhaseJMoogURappoldGRauchARopersHHvon SpiczakSTönniesHVilleneuveNVillardLZabelBZenkerMLaubeBReisAWieczorekDVan MaldergemLKutscheK: Mutations in GRIN2A and GRIN2B encoding regulatory subunits of NMDA receptors cause variable neurodevelopmental phenotypes. *Nat Genet*.2010;42(11) : 10.1038/ng.677 1021-6 10.1038/ng.677 20890276

[2] BurnashevNSzepetowskiP: NMDA receptor subunit mutations in neurodevelopmental disorders. *Curr Opin Pharmacol*.2015;20: 10.1016/j.coph.2014.11.008 73-82 10.1016/j.coph.2014.11.008 25498981

[3] TakasakiYKoideTWangCKimuraHXingJKushimaIIshizukaKMoriDSekiguchiMIkedaMAizawaMTsurumaruNIwayamaYYoshimiAAriokaYYoshidaMNomaHOya-ItoTNakamuraYKunimotoSAleksicBUnoYOkadaTUjikeHEgawaJKuwabaraHSomeyaTYoshikawaTIwataNOzakiN: Mutation screening of GRIN2B in schizophrenia and autism spectrum disorder in a Japanese population. *Sci Rep*.2016;6: 10.1038/srep33311 33311 10.1038/srep33311 27616045PMC5018849

[4] SwangerSAChenWWellsGBurgerPBTankovicABhattacharyaSStrongKLHuCKusumotoHZhangJAdamsDRMillichapJJPetrovskiSTraynelisSFYuanH: Mechanistic Insight into NMDA Receptor Dysregulation by Rare Variants in the GluN2A and GluN2B Agonist Binding Domains. *Am J Hum Genet*.2016;99(6) : 10.1016/j.ajhg.2016.10.002 1261-1280 10.1016/j.ajhg.2016.10.002 27839871PMC5142120

[5] SerrazBGrandTPaolettiP: Altered zinc sensitivity of NMDA receptors harboring clinically-relevant mutations. *Neuropharmacology*.2016;109: 10.1016/j.neuropharm.2016.06.008 196-204 10.1016/j.neuropharm.2016.06.008 27288002

[6] ZhengFGingrichMBTraynelisSFConnPJ: Tyrosine kinase potentiates NMDA receptor currents by reducing tonic zinc inhibition. *Nat Neurosci*.1998;1(3) : 10.1038/634 185-91 10.1038/634 10195142

# Peer review: Referee response for version 1

**DOI:** 10.21956/wellcomeopenres.11843.r21096

**Published:** 2017-04-06

**Authors:** Ian R. Mellor, Helen Knight

**Affiliations:** 1 School of Life Sciences, University of Nottingham, Nottingham, UK; 2 University of Nottingham, Nottingham, UK

Recent large scale exome sequencing studies of disease and biomedically relevant quantitative traits have revealed a high frequency of rare coding loss of function mutations and amino acid changing missense variants across the genome ^1^^,^^2^.  It is now also recognised that such rare coding variants may not necessarily have a functional impact. One such category of coding variant commonly known as Variants of Unknown Significance (VUS), are typically composed of missense mutations (amino acid substitutions) and are often carried in a heterozygous state. Whether such variants have a functional effect, and potentially could be pathogenic, depend on a number of factors including the mutated gene and gene product, genetic sequence context of the variant, and, where in the encoded protein the amino acid substitution occurs. However, certain families of genes have consistently been indicated to contain genetic variation associated with specific diseases. One such family of genes encode for glutamate neurotransmitter receptor subunits and have been associated with risk for developing a variety of brain diseases.

 The manuscript describes an *in vitro *functional study of a missense variant within the NMDA type glutamate receptor subunit, GluN2A (gene *GRIN2A), *which had originally been identified in the heterozygous state in an individual with epilepsy and/or abnormal EEG and reported by Endele et al., 2010. The variant was reported to be inherited and not arising *de novo, *which is often considered to have a higher probability of being a pathogenic mutation. Mutations within *GRIN2A *have previously been reported to cause a range of epilepsy syndromes consistent with an inherited autosomal dominant Mendelian manner ^3^^,^^4^ and also to contribute to risk for common complex neurodevelopmental diseases such as severe intellectual disability ^5^. The question of whether this VUS has a functional effect is indeed very pertinent.

 We find that the manuscript is written well with the aims and methods appropriately detailed. We have some comments regarding the experimental work and some suggestions for inclusion in the introduction and discussion sections, which we hope will contribute to the quality of the manuscript.

**Abstract**

 The abstract needs to include the human gene name ( *GRIN2A*) and that rare variants have been identified within NMDA subunit genes in individuals with neurodevelopmental disorders as well as in unaffected individuals.

**Introduction**

 It would be useful to have more of an overview of how genetic variation within *GRIN2A* is thought to contribute to brain disease, i.e. genotype-phenotype relationships (e.g. type of mutations, mode of inheritance, ‘causative’ for disease or associated with increased risk of disease).

 It should be stated in the introduction that *in silico* tools had been used to predict the function consequence of this variant (as presented by Endele *et al.,* 2010,  supplementary Table 2) and that such prediction tools including others not used by Endele *et al., *indicate that this variant is predicted to be consistently ‘possibly damaging’ as opposed to benign. This provides good justification to study the variant. It is also probably worth pointing out the severity of the amino acid substitution in the studied variant, i.e. it is a conservative change.

 Where discussing NMDARs as a “molecular coincidence detector”, it might be worth mentioning the requirement for glycine as co-agonist to glutamate too.

**Methods**

 The methods were very well explained and are entirely repeatable.

**Results**

 The first part of the results section shows the sensitivity of the NMDARs containing WT or mutated GluN2A to Mg ^2+^. The chosen concentration of Mg ^2+^ is indeed around the expected physiological concentration, but because it is more-or-less a saturating inhibitory concentration it would be harder to pick up any changes in sensitivity. Repeating the experiment with a lower concentration of Mg ^2+^ or better still obtaining an IC _50_ would give more substance to the claim that this property is unchanged. The same is true for the experiments with transfected neurons where inhibition is very close to 100%.

 The methods section states that oocytes were clamped at several holding potentials but data is only shown for -60 mV. It would strengthen the claim that Mg ^2+^ sensitivity is unchanged if data for other holding potential is shown and/or discussed. Small changes to the voltage-dependence of inhibition could be responsible for defective physiological function of NMDARs.

 The responses of transfected neurons to NMDA in Fig. 2A-B may imply small alterations to desensitisation properties. Was there any measurable difference?

 In the single channel study, was there any difference in the mean closed time, and what was the outcome of the dwell time (open and closed) analysis mentioned in the methods?

**Discussion**

 In paragraph 2, more details are needed to explain why there may have been differences between the present findings and those previously reported for mutations found to have an impact on NMDA subunit properties. Indeed, with the breadth of type of mutations identified for different brain diseases and their mode of inheritance (loss of function, gain of function, het state) would you expect the same in terms of observable change of function?

 The authors state that this variant has been identified in two individuals out of 60,706 in the ExAC database and hence the authors suggest this provides additional evidence that this variant is not pathogenic and the occurrence in an individual with epilepsy was coincidental. However, the ExAC consortium includes genome data from individuals belonging to disease cohort studies as well as healthy ‘control’ individuals. All that is currently published about these two individuals who have this variant is that they do not have severe paediatric disease. It may be that these individuals belong to disease cohorts and have a diagnosis of a brain disease including neurodevelopmental disorders.

 It is quite possible that the genotype-phenotype relationship is not as straight forward as the original hypothesis, i.e. that this variant in isolation has a functional effect and that the effect on subunit properties is directly through an amino acid change. For example, several coding variants are thought to have an effect in tandem with other coding mutations (compound heterozygous mutations). Alternatively, other genomic/epigenetic mechanisms may be disrupted by this variant and underlie transcription and translation processes which affect subunit abundance.

We have read this submission. We believe that we have an appropriate level of expertise to confirm that it is of an acceptable scientific standard, however we have significant reservations, as outlined above.

## References

[1] UK10K ConsortiumWalterKMinJLHuangJCrooksLMemariYMcCarthySPerryJRXuCFutemaMLawsonDIotchkovaVSchiffelsSHendricksAEDanecekPLiRFloydJWainLVBarrosoIHumphriesSEHurlesMEZegginiEBarrettJCPlagnolVRichardsJBGreenwoodCMTimpsonNJDurbinRSoranzoN: The UK10K project identifies rare variants in health and disease. *Nature*.2015;526(7571) : 10.1038/nature14962 82-90 10.1038/nature14962 26367797PMC4773891

[2] LekMKarczewskiKMinikelESamochaKBanksEFennellTO’Donnell-LuriaAWareJHillACummingsBTukiainenTBirnbaumDKosmickiJDuncanLEstradaKZhaoFZouJPierce-HoffmanEBerghoutJCooperDDeflauxNDePristoMDoRFlannickJFromerMGauthierLGoldsteinJGuptaNHowriganDKiezunAKurkiMMoonshineANatarajanPOrozcoLPelosoGPoplinRRivasMRuano-RubioVRoseSRuderferDShakirKStensonPStevensCThomasBTiaoGTusie-LunaMWeisburdBWonHYuDAltshulerDArdissinoDBoehnkeMDaneshJDonnellySElosuaRFlorezJGabrielSGetzGGlattSHultmanCKathiresanSLaaksoMMcCarrollSMcCarthyMMcGovernDMcPhersonRNealeBPalotieAPurcellSSaleheenDScharfJSklarPSullivanPTuomilehtoJTsuangMWatkinsHWilsonJDalyMMacArthurD: Analysis of protein-coding genetic variation in 60,706 humans. *Nature*.2016;536(7616) : 10.1038/nature19057 285-291 10.1038/nature19057 PMC501820727535533

[3] CarvillGLReganBMYendleSCO'RoakBJLozovayaNBruneauNBurnashevNKhanACookJGeraghtyESadleirLGTurnerSJTsaiMHWebsterROuvrierRDamianoJABerkovicSFShendureJHildebrandMSSzepetowskiPSchefferIEMeffordHC: GRIN2A mutations cause epilepsy-aphasia spectrum disorders. *Nat Genet*.2013;45(9) : 10.1038/ng.2727 1073-6 10.1038/ng.2727 23933818PMC3868952

[4] LemkeJRLalDReinthalerEMSteinerINothnagelMAlberMGeiderKLaubeBSchwakeMFinsterwalderKFrankeASchilhabelMJähnJAMuhleHBoorRVan PaesschenWCaraballoRFejermanNWeckhuysenSDe JonghePLarsenJMøllerRSHjalgrimHAddisLTangSHughesEPalDKVeriKVaherUTalvikTDimovaPGuerrero LópezRSerratosaJMLinnankiviTLehesjokiAERufSWolffMBuerkiSWohlrabGKroellJDattaANFiedlerBKurlemannGKlugerGHahnAHaberlandtDEKutzerCSpernerJBeckerFWeberYGFeuchtMSteinböckHNeophythouBRonenGMGruber-SedlmayrUGeldnerJHarveyRJHoffmannPHermsSAltmüllerJToliatMRThieleHNürnbergPWilhelmCStephaniUHelbigILercheHZimprichFNeubauerBABiskupSvon SpiczakS: Mutations in GRIN2A cause idiopathic focal epilepsy with rolandic spikes. *Nat Genet*.2013;45(9) : 10.1038/ng.2728 1067-72 10.1038/ng.2728 23933819

[5] de LigtJWillemsenMHvan BonBWKleefstraTYntemaHGKroesTVulto-van SilfhoutATKoolenDAde VriesPGilissenCdel RosarioMHoischenASchefferHde VriesBBBrunnerHGVeltmanJAVissersLE: Diagnostic exome sequencing in persons with severe intellectual disability. *N Engl J Med*.2012;367(20) : 10.1056/NEJMoa1206524 1921-9 10.1056/NEJMoa1206524 23033978

# Peer review: Referee response for version 1

**DOI:** 10.21956/wellcomeopenres.11843.r21350

**Published:** 2017-04-05

**Authors:** Paul L. Chazot

**Affiliations:** 1 School of Biological and Biomedical Science, Durham University, Durham, UK

This is a well written, straight-forward study which has been performed well.

 Explanation of why this particular mutation was selected over the many choices in key regions of the GluN2A subunit would be useful.

 As the authors allude to, this not a comprehensive study of the short and long term functional property changes which may be linked to the mutation.

I have read this submission. I believe that I have an appropriate level of expertise to confirm that it is of an acceptable scientific standard.
